# Supplementary material for: Intravenous treatment of choroidal neovascularization by photo-targeted nanoparticles
Source: Nat Commun. 2019 Feb 18;10:804. doi: 10.1038/s41467-019-08690-4 (PMC6379485; doi:10.1038/s41467-019-08690-4)
Supplement: Supplementary file 1 — Supplementary Information [file 41467_2019_8690_MOESM1_ESM.pdf]

Supplementary Information

Intravenous treatment of choroidal neovascularization by photo-targeted nanoparticles

Yanfei Wang[a], Chi-Hsiu Liu[b], Tianjiao Ji[a], Manisha Mehta[a], Weiping Wang[a], Elizabeth Marino[a], Jing Chen[b], and Daniel S. Kohane[a,*]

[a]Laboratory for Biomaterials and Drug Delivery, Department of Anesthesiology, Division of Critical Care Medicine, Boston Children's Hospital, Harvard Medical School, 300 Longwood Avenue, Boston, Massachusetts 02115, United States
[b]Department of Ophthalmology, Boston Children's Hospital, Harvard Medical School, Boston, MA 02115
* daniel.kohane@childrens.harvard.edu

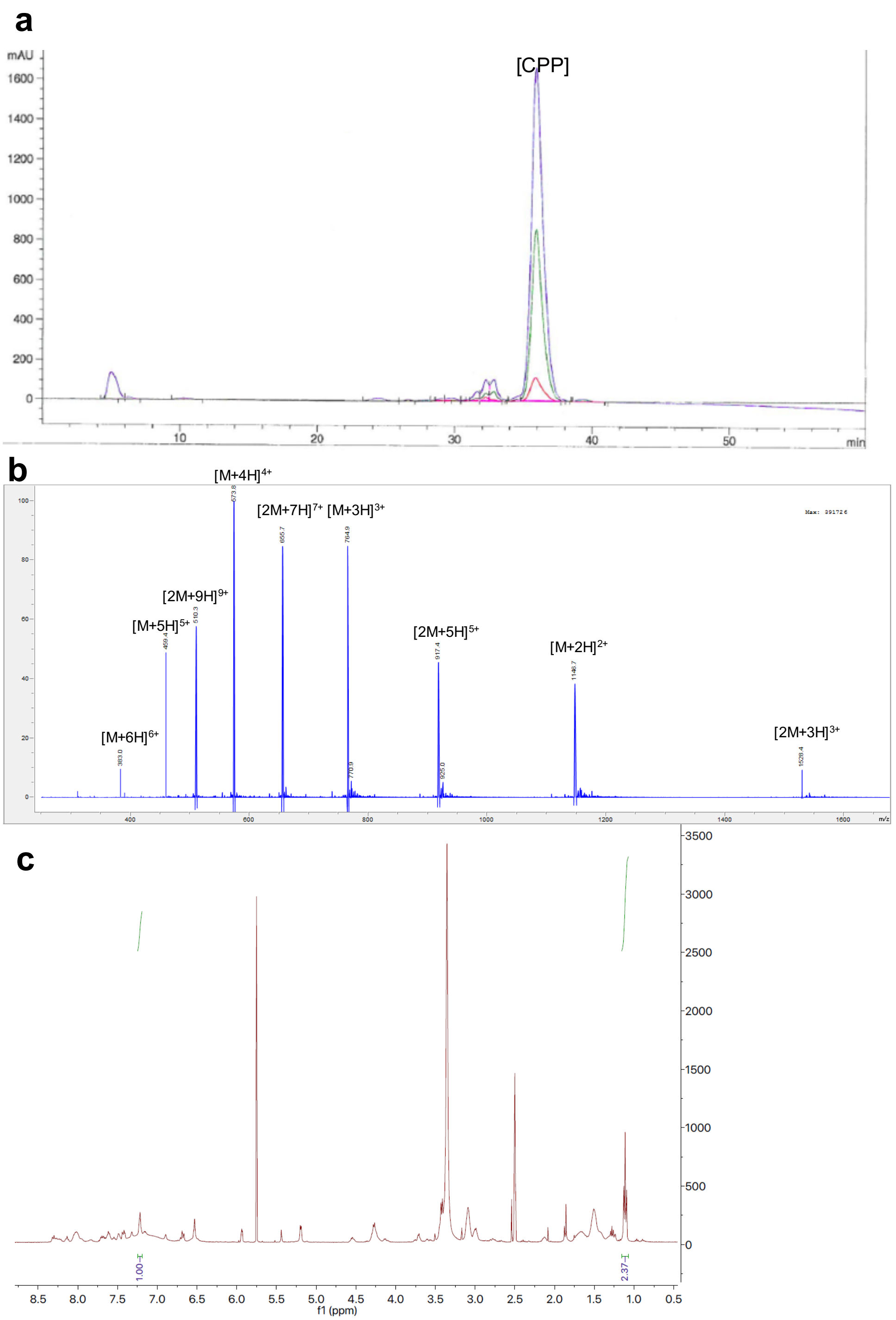

Supplementary Figure 1. Characterization of [CPP]. a, HPLC of purified [CPP], monitored at 210nm (blue line), 280nm (red line) and 385nm (green line). b, Electrospray ionization mass spectroscopy (ESI-MS) spectrum of [CPP](m/z = 2292.2), using positive scan mode on an Agilent 6130 Single Quad LCMS instrument. MW of unmodified CPP is 1746.1. c, NMR spectrum of [CPP], in DMSO-d_6.

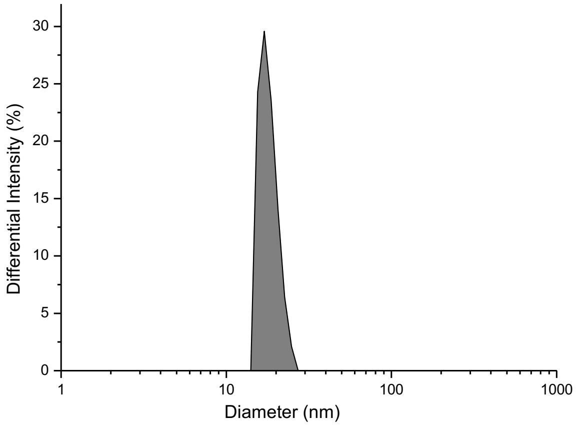

Supplementary Figure 2. Representative number-weighted size distribution of NP-[CPP] in PBS, determined by dynamic light scattering (DLS).

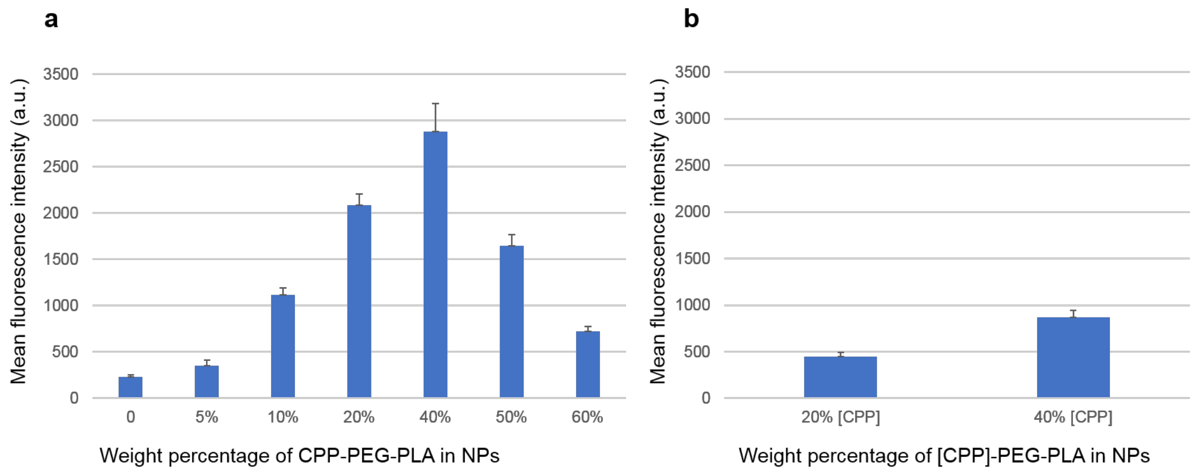

Supplementary Figure 3. Quantification of flow cytometric analyses of HUVEC uptake of nanoparticles containing different percentages of peptide-polymer conjugate. a, Effect of percentage of CPP-PEG-PLA in nanoparticles on cell uptake (mean fluorescent intensity of cells). b, Effect of percentage of [CPP]-PEG-PLA in nanoparticles on cell uptake. Data are means ± SD (n = 4).

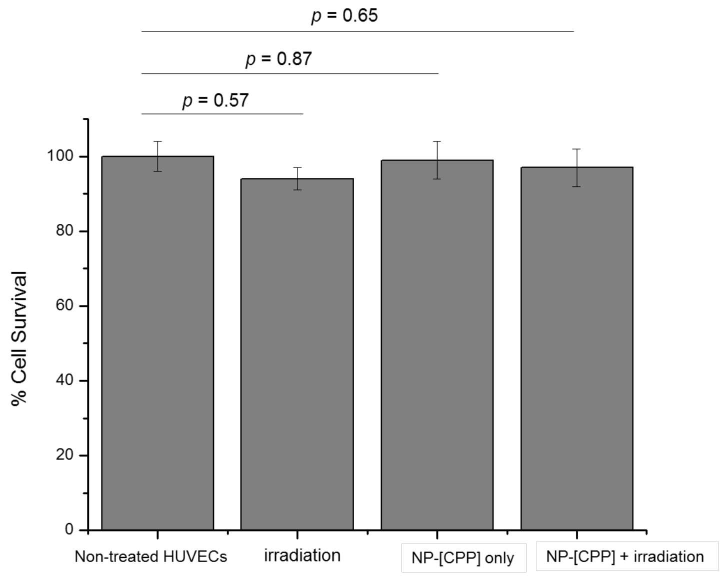

Supplementary Figure 4. Survival rates determined by MTS assay of HUVECs after irradiation (400 nm at 50 mW/cm^{-2} for 1 min, at the beginning of incubation) and/or overnight incubation with 0.5 mg/mL NP-[CPP], with non-treated HUVECs as controls. Data are means ± SD (n = 4).

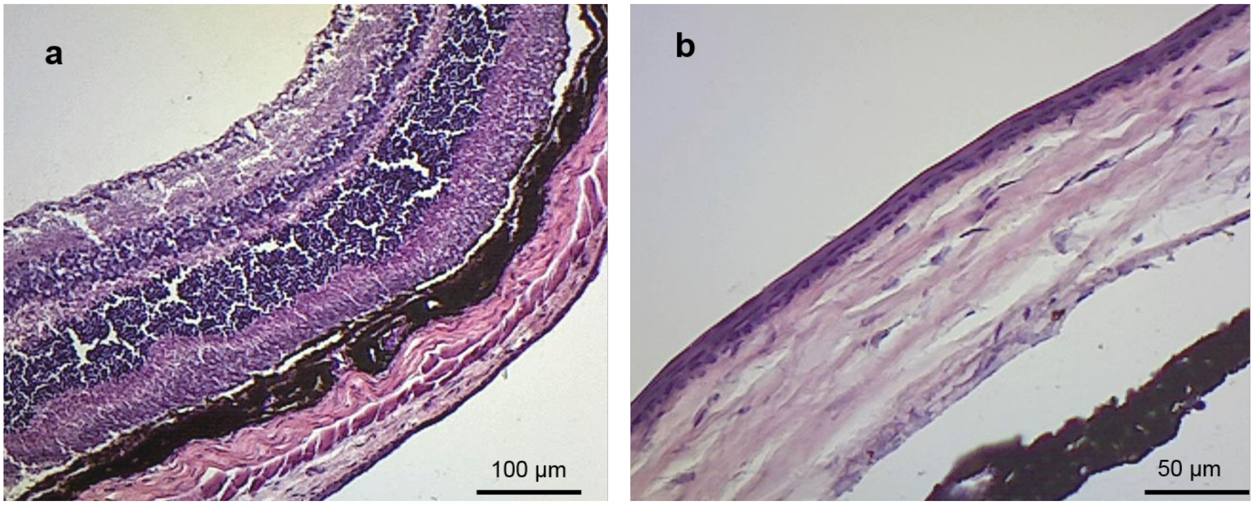

Supplementary Figure 5. Representative histology of untreated normal eyes (Hematoxylin and eosin). a, Normal retinal histology. b, Normal corneal histology.

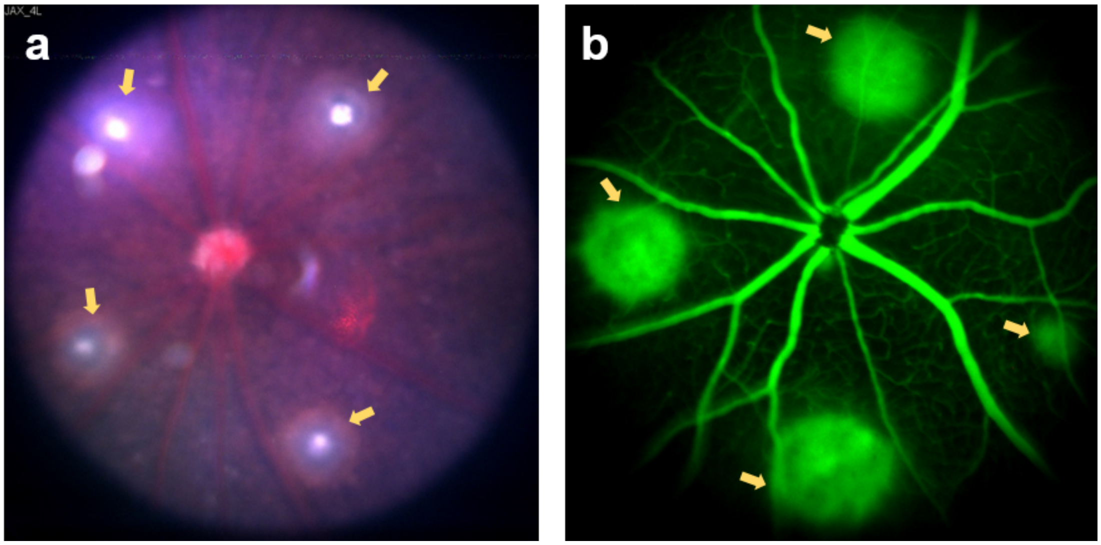

Supplementary Figure 6. Representative images of laser-induced mouse CNV model. a, The development of vapor bubbles (arrows) in Bruch's membrane after laser irradiation. b, Fluorescein angiograph of the mouse retina taken with a Micron IV imaging system 6 days after laser injury. Mice were injected intraperitoneally with Fluorescein AK-FLUOR® (100mg/ml) at 100μg/g (body weight). Four areas of neovascularization are demonstrated (arrows).

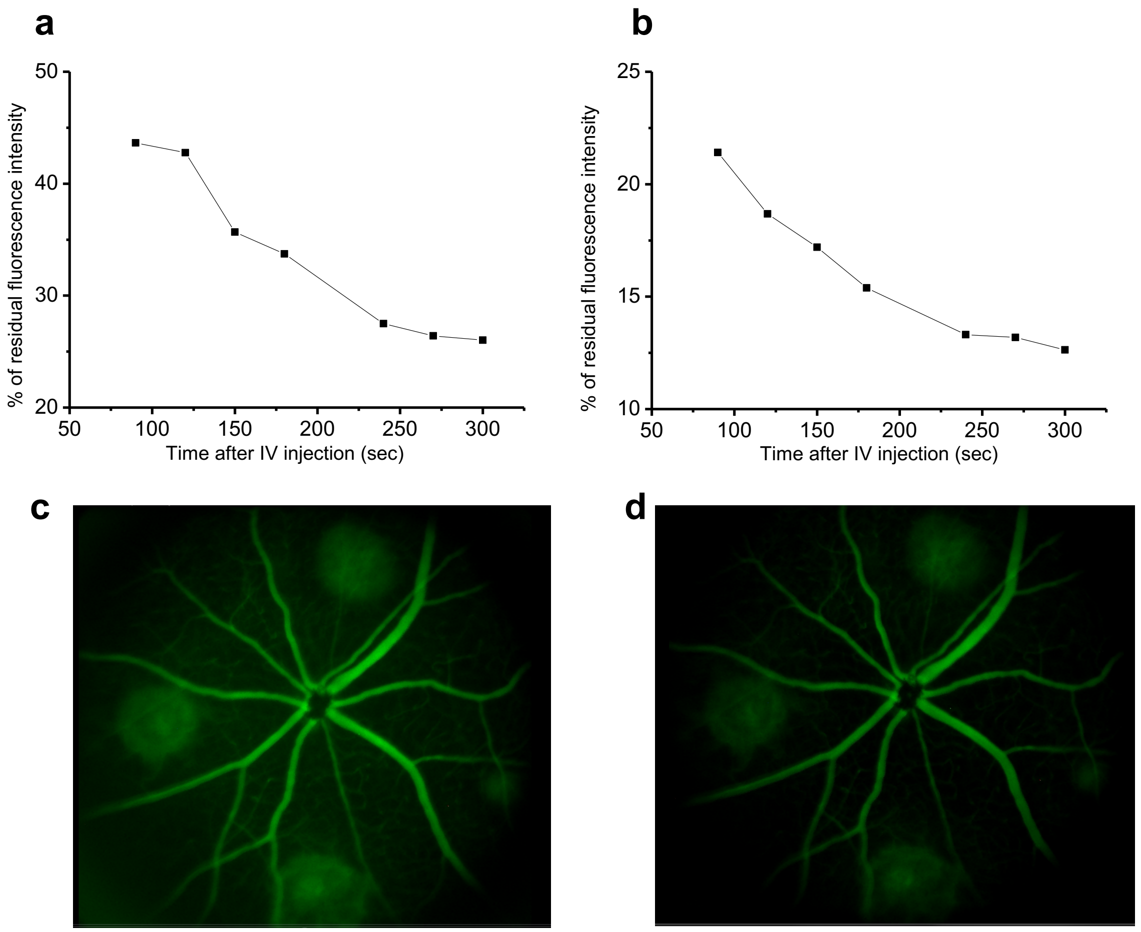

Supplementary Figure 7. Fluorescence intensity in the retina. Quantification in a representative single animal of the average fluorescent intensity in (a) retinal blood vessels and (b) laser-induced lesions, after IV injection of fluorescently labeled nanoparticles. (c, d) Representative images at (c) 100s and (d) 300s after NP-AMF injection.

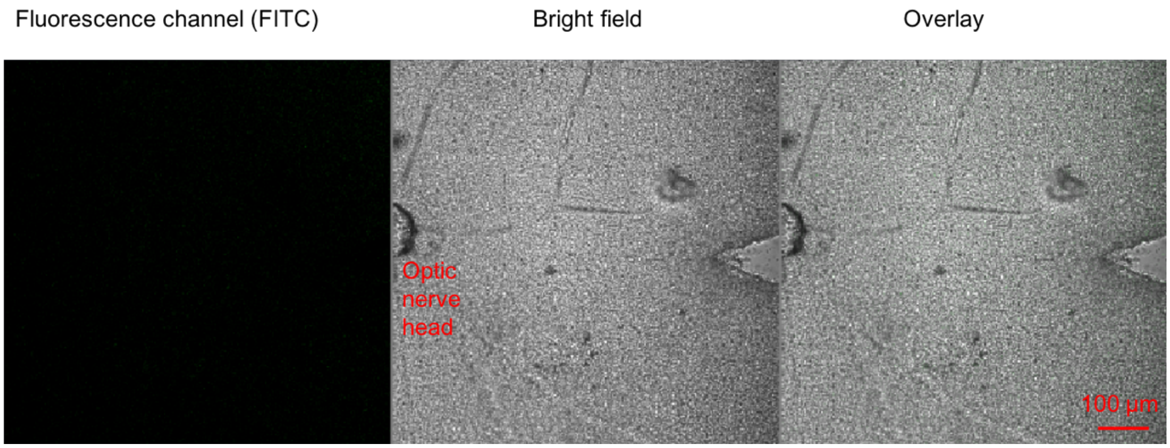

Supplementary Figure 8. A representative (of 10) fluorescent image of retinal flat-mounts. Mice were injected with fluorescently labeled nanoparticles via tail vein 24 hrs before these images were taken.

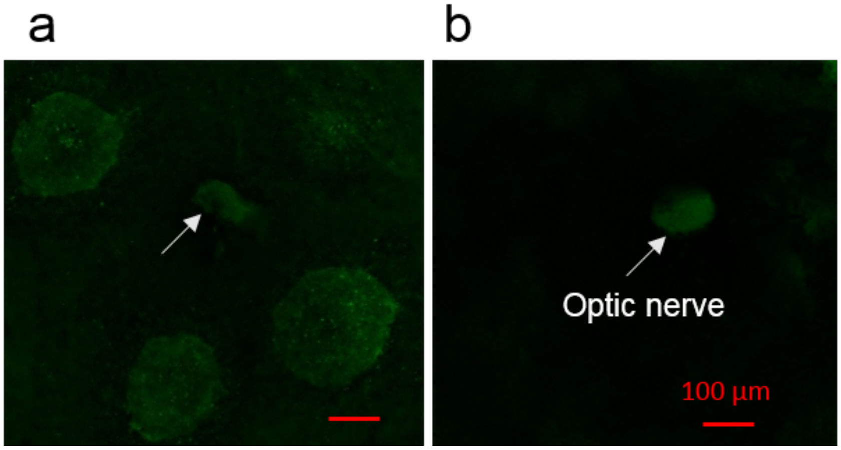

Supplementary Figure 9. Comparison of representative (of 4 eyes) choroidal flat-mounts of a CNV eye and a normal eye, 24 hrs after NP-AMF injection. a, Fluorescence in four lesions was observed in choroidal flat-mounts of a CNV eye and autofluorescence in the optic disc (arrow). b, Minimal fluorescence was observed in choroidal flat-mounts of a healthy eye, aside from autofluorescence of the optic disc.

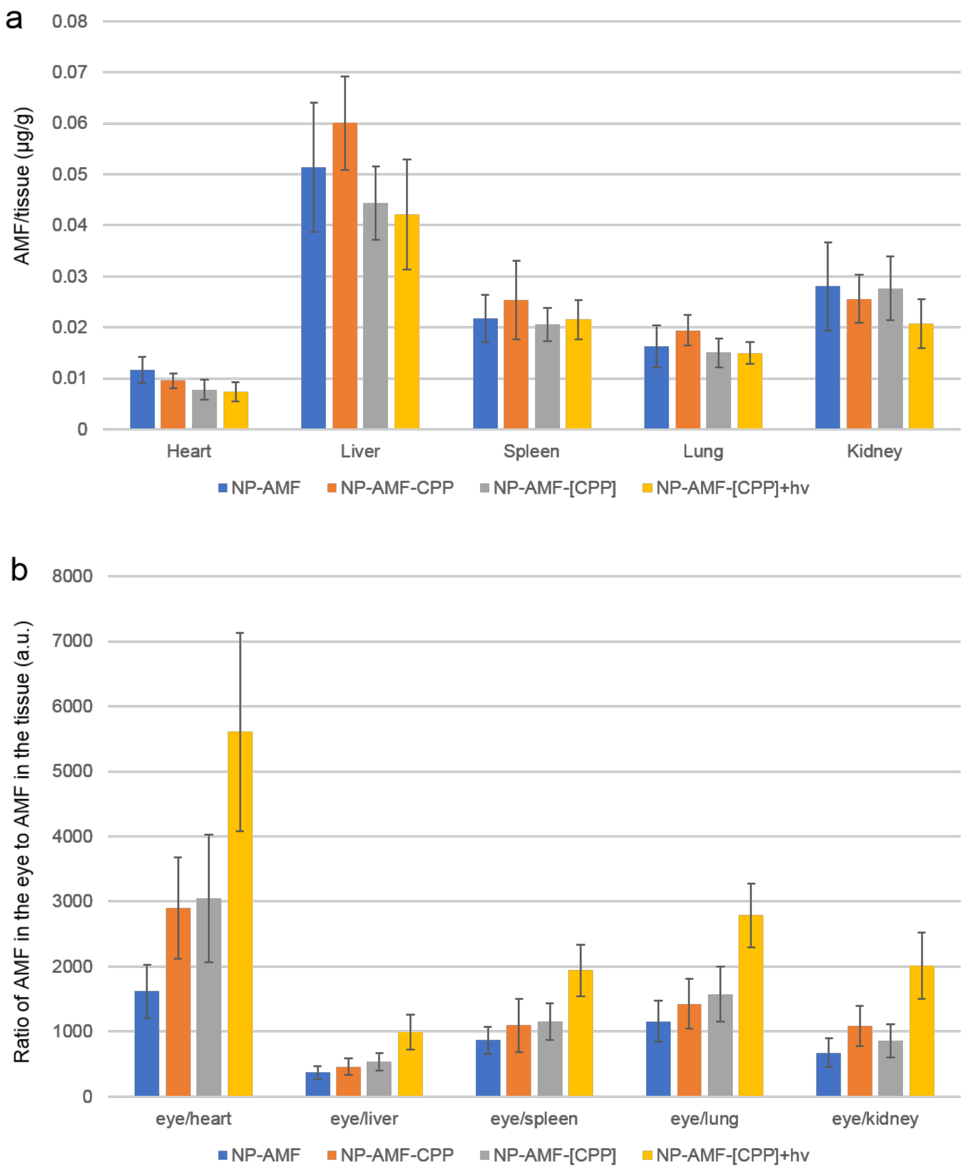

Supplementary Figure 10. Biodistribution of injected formulations in mice 24 h after intravenous injection. a, Effect of treatment group on AMF per gram of tissue harvested. b, Effect of treatment group on ratio of AMF in the eye (from data in Figure 4b) to AMF in tissue (data in figure S10a). Data are means ± SD (n = 4).

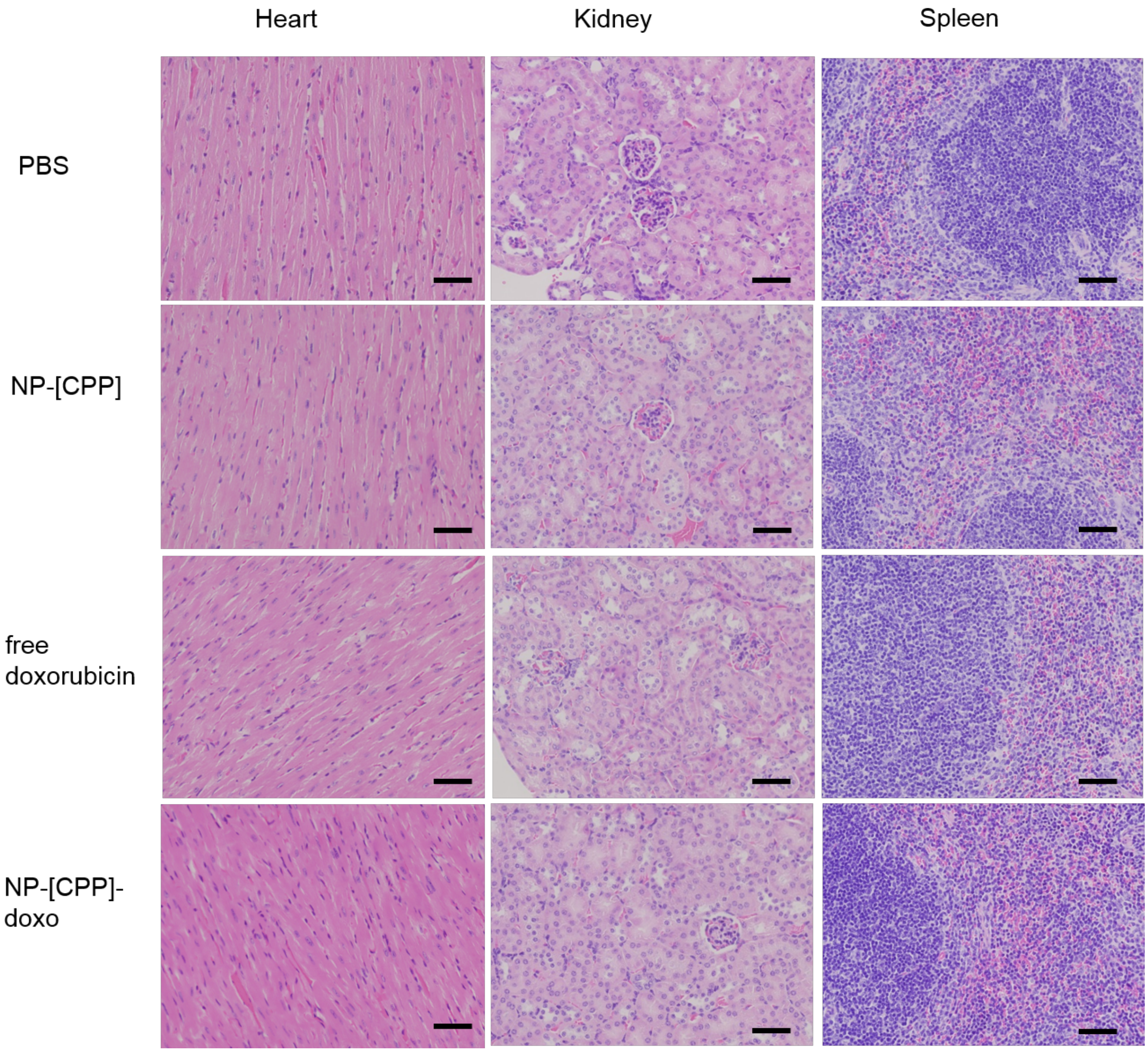

Supplementary Figure 11. Histology of heart, kidney, and spleen of control group (PBS), and treated groups (NP-[CPP], free doxorubicin, NP-[CPP]-doxo (Hematoxylin and eosin). (The scale bar is 50 µm).

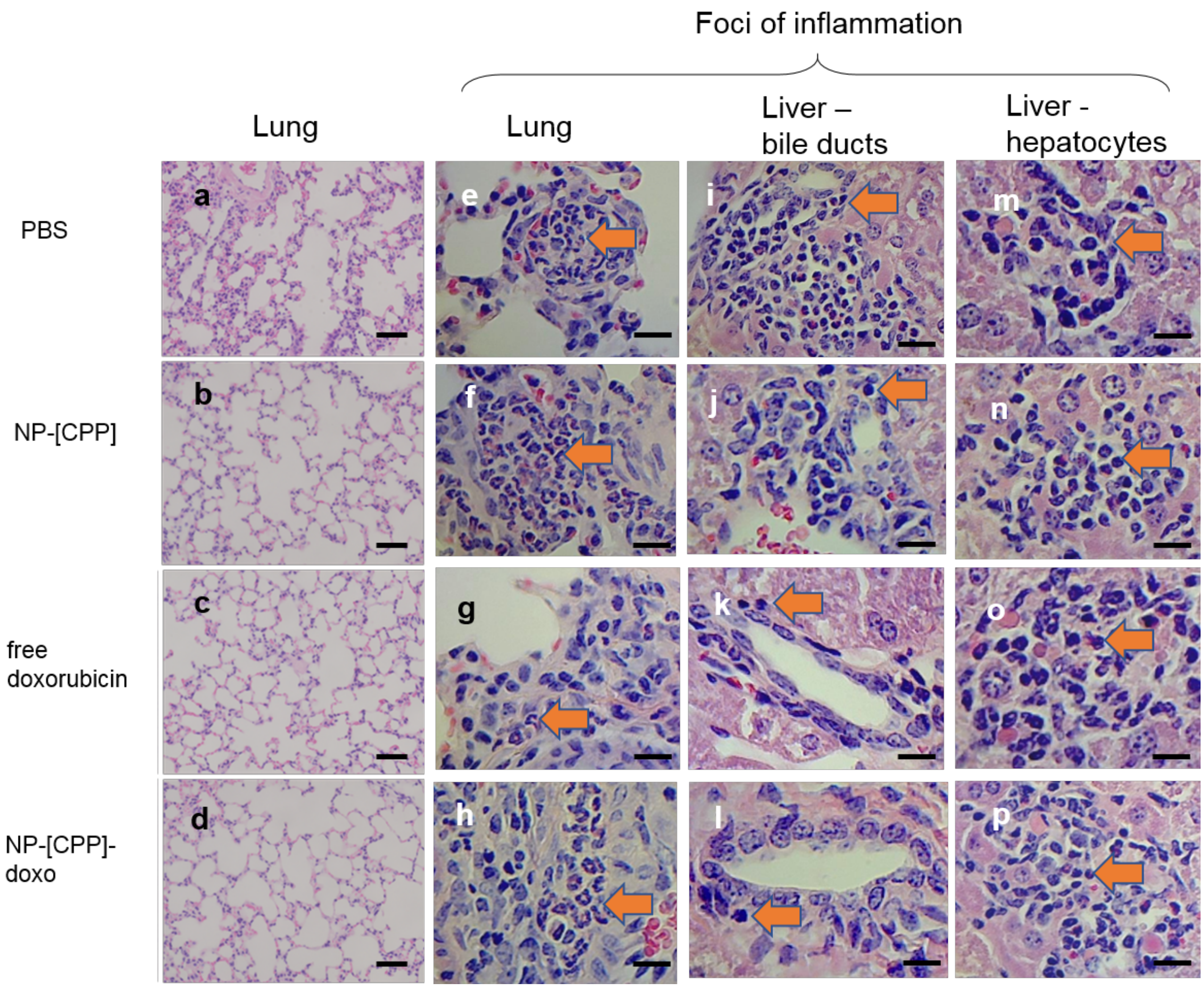

Supplementary Figure 12. Representative photomicrographs of hematoxylin-eosin-stained sections of lung and liver in animals administered PBS, or treated with NP-[CPP], free doxorubicin, or NP-[CPP]-doxo, after one week of treatment. Scale bar is 50 µm in a-d, 25 µm in E-P. a-d: Predominantly normal lung histology seen in all groups. e-h: Lung: Foci of acute interstitial inflammation (neutrophils: arrow). i-l: Liver: Foci of chronic inflammation (lymphocytes: arrow) in the portal areas surrounding the bile ducts. m-p: Liver: Foci of lobulitis: lymphocytes (arrow) surrounding hepatocytes.

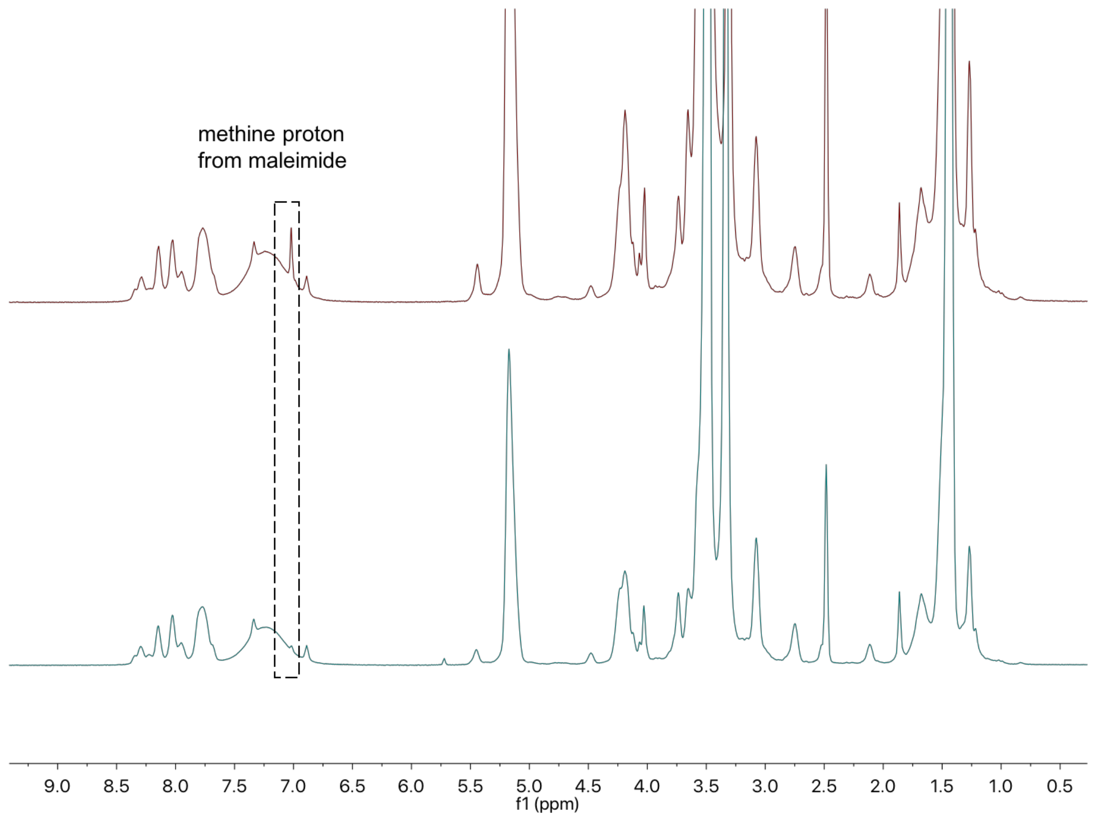

Supplementary Figure 13. ^{1}H NMR spectrum (in DMSO-d_6) of the reaction mixture of maleimide-PEG-PLA and [CPP] before (top) and after (bottom) the conjugation reaction, with the methine proton peak from maleimide highlighted in the dotted rectangle.

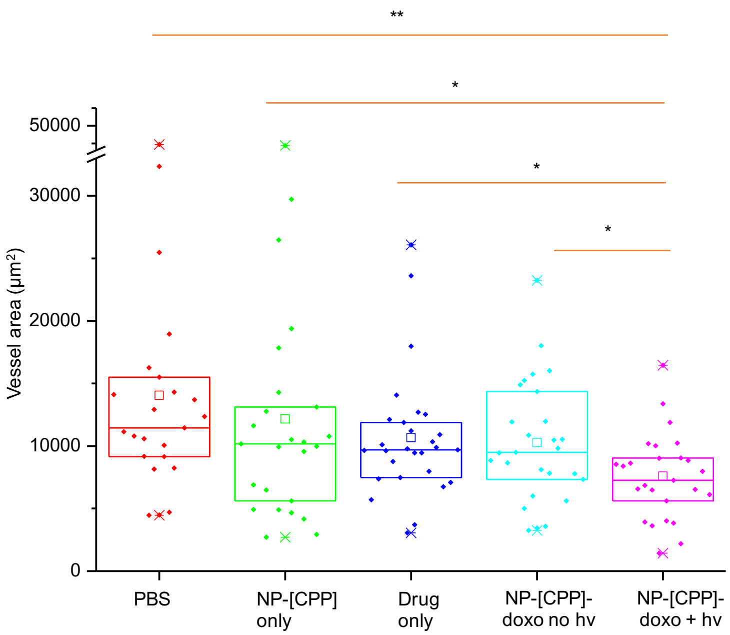

Supplementary Figure 14. Effect of treatment groups on vessel area. Dots are the individual data points. The box displays the median values with 25th and 75th percentiles and the square is the mean value. n = 28 lesions. *$P < 0.05$, **$P < 0.005$.

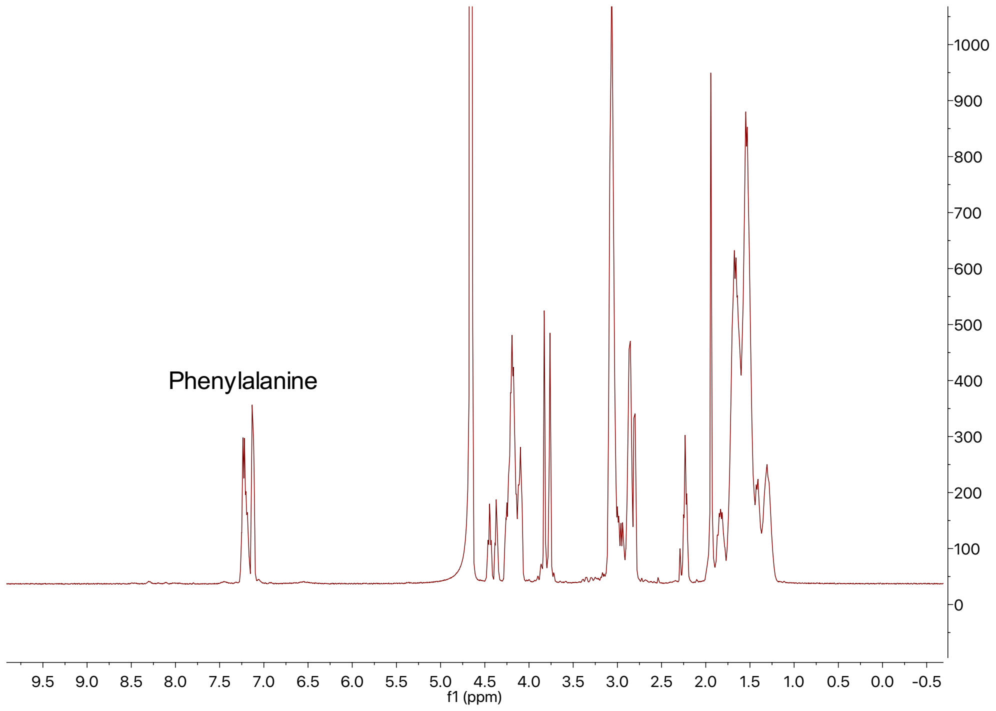

Supplementary Figure 15. Full NMR spectrum of unmodified CPP.

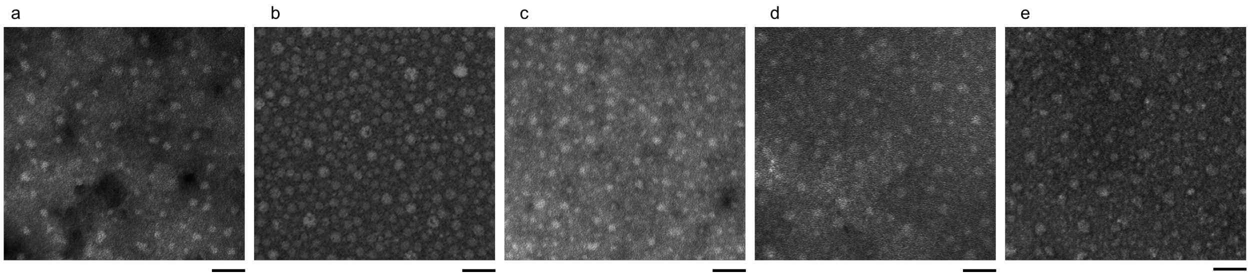

Supplementary Figure 16. TEM images of all NPs: NP (a), NP-[CPP] (b), NP-[CPP]+h*ν* (c), NP-CPP (d), NP-[CPP]-doxo (e). The scale bar is 50 nm.

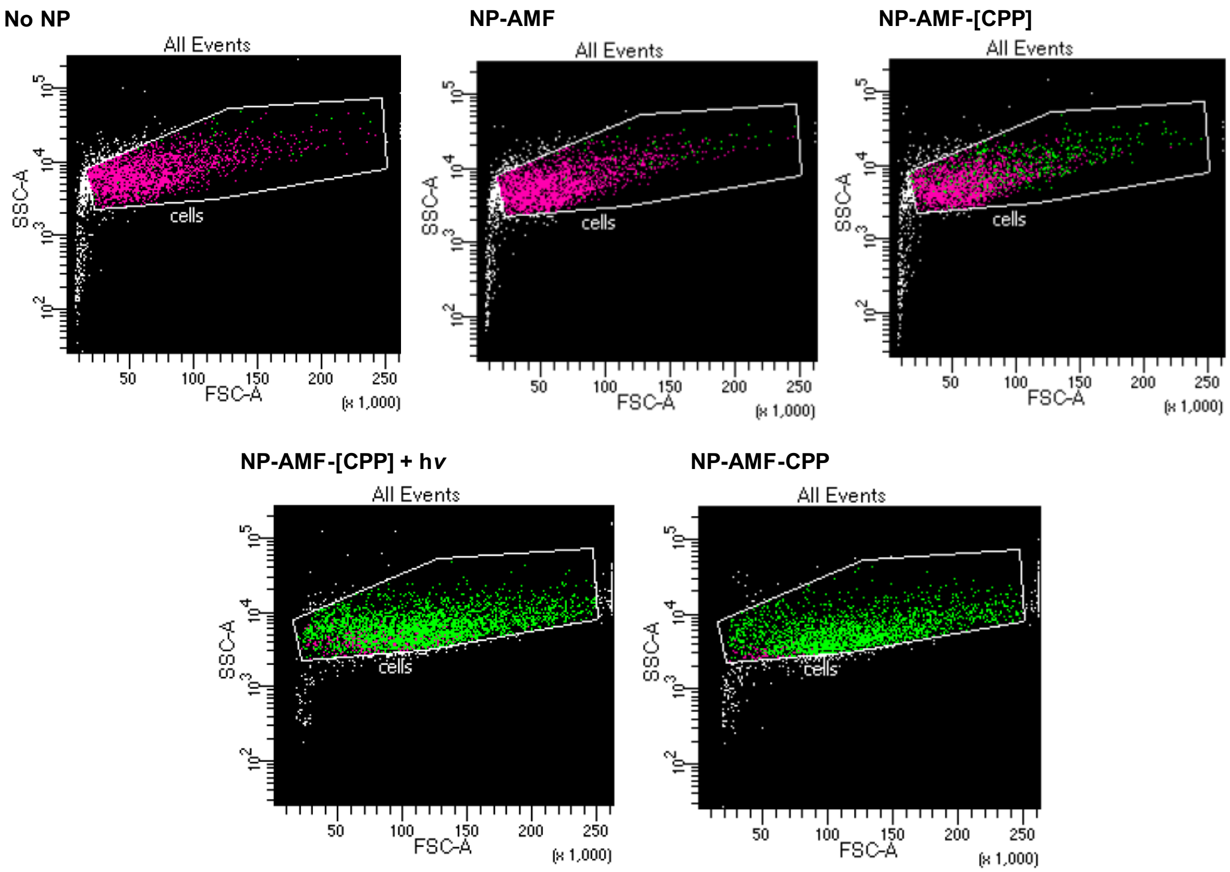

Supplementary Figure 17. Representative FSC (Forward SCatter)-SSC (Side SCatter) (from flow cytometry)data. The purple color labels cells that are not FITC positive, while the green color labels FITC-positive cells. The size (from FSC) and granularity (from SSC; this metric would detect breakdown and/or aggregation of cells) of HUVECs remained the same for all groups.

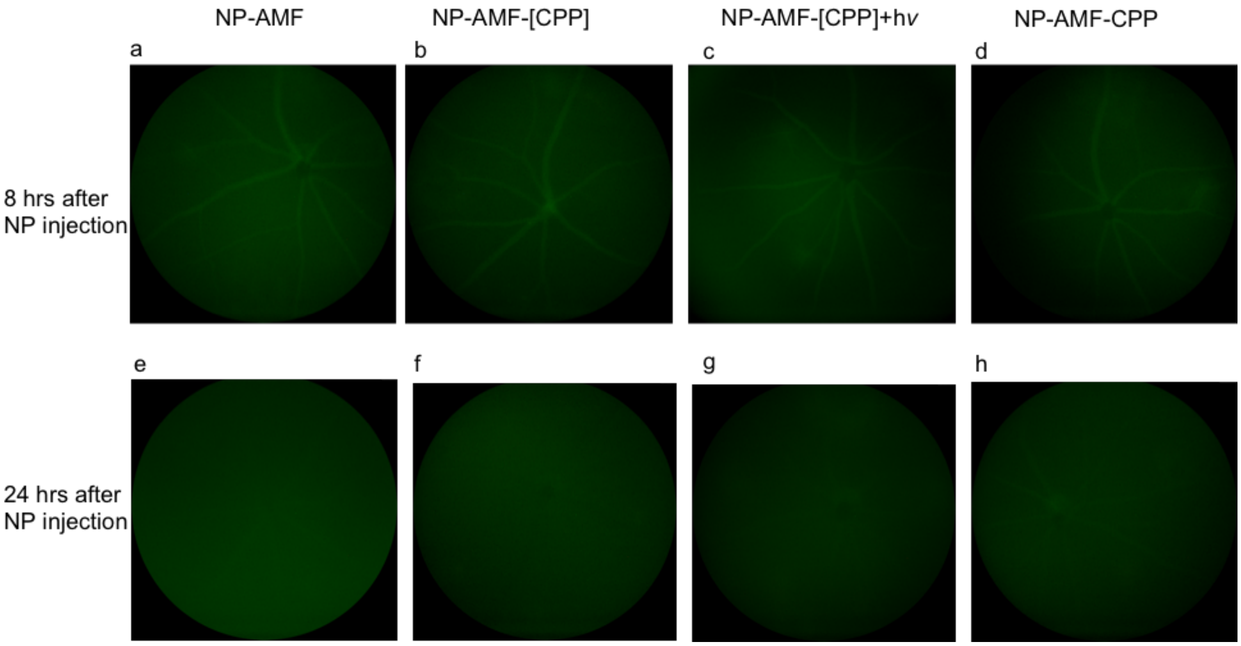

Supplementary Figure 18. *In vivo* fluorescence images of mouse fundus 8hrs (a-d) and 24 hrs (e-h) after IV injection of nanoparticles. Faint fluorescence in blood vessels and lesions can be seen at 8 h.

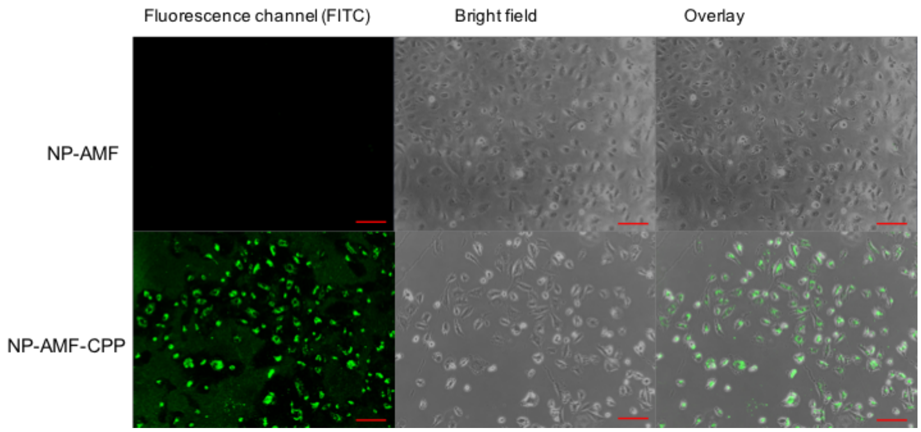

Supplementary Figure 19. Representative confocal microscopic images of HUVEC uptake of nanoparticles. The scale bar is 100 μm.

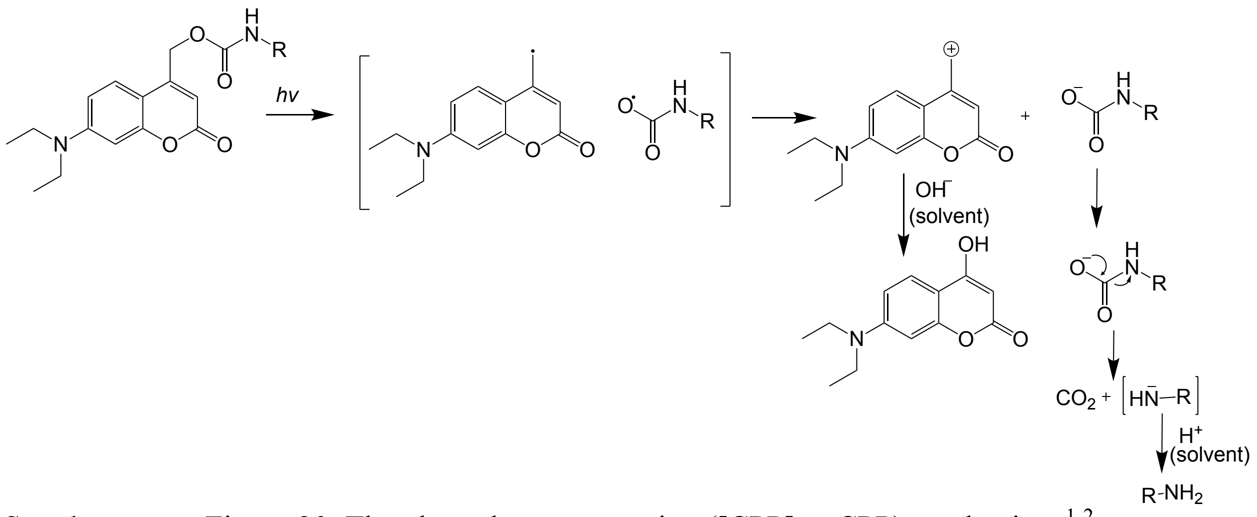

Supplementary Figure 20. The photocleavage reaction ([CPP] to CPP) mechanism.[1-2]

Supplementary Table 1. Mean, standard deviation (SD), coefficient of variation, and standard error of the mean (SEM) of data in Figure 5b.

	Mean	SD	SEM*
PBS	14042	9242	1927
NP-[CPP] only	12166	9517	1903
Drug only	10675	5021	948
NP-[CPP]-doxo	10278	4802	924
NP-[CPP]-doxo + hv	7580	3416	669

*The SEM is generally used in the literature on this subject as the metric of variation from the mean.[3] We have followed that practice in this manuscript in order to facilitate comparison of our results to that of other investigators. We are providing the SD here for completeness.

Supplementary Table 2. Hydrodynamic diameter and zeta potential of NP, NP-[CPP], NP-[CPP]+hv, NP-CPP and NP-[CPP]-doxo.

	Hydrodynamic diameter (nm)	Zeta potential (mV)
NP	18.4 ± 2.6	-6.2 ± 1.0
NP-[CPP]	19.0 ± 2.0	-4.1 ± 0.8
NP-[CPP]+ hv	20.1 ± 3.2	10.5 ± 1.4
NP-CPP	18.8 ± 2.9	9.8 ± 1.6
NP-[CPP]-doxo	29.4 ± 4.1	-1.0 ± 0.2

Supplementary References

1. Eckardt, T.; Hagen, V.; Schade, B.; Schmidt, R.; Schweitzer, C.; Bendig, J., Deactivation Behavior and Excited-State Properties of (Coumarin-4-yl)methyl Derivatives. 2. Photocleavage of Selected (Coumarin-4-yl)methyl-Caged Adenosine Cyclic 3‘,5‘-Monophosphates with Fluorescence Enhancement. *The Journal of Organic Chemistry* **2002,** *67* (3), 703-710.
2. Klán, P.; Šolomek, T.; Bochet, C. G.; Blanc, A.; Givens, R.; Rubina, M.; Popik, V.; Kostikov, A.; Wirz, J., Photoremovable Protecting Groups in Chemistry and Biology: Reaction Mechanisms and Efficacy. *Chemical Reviews* **2013,** *113* (1), 119-191.
3. Liu, C.-H.; Sun, Y.; Li, J.; Gong, Y.; Tian, K. T.; Evans, L. P.; Morss, P. C.; Fredrick, T. W.; Saba, N. J.; Chen, J., Endothelial microRNA-150 is an intrinsic suppressor of pathologic ocular neovascularization. *Proceedings of the National Academy of Sciences* **2015,** *112* (39), 12163-12168.
