# Supplementary material for: Intravenous treatment of choroidal neovascularization by photo-targeted nanoparticles
Source: Nat Commun. 2019 Feb 18;10:804. doi: 10.1038/s41467-019-08690-4 (PMC6379485; doi:10.1038/s41467-019-08690-4)
Supplement: Supplementary file 3 — Description of Additional Supplementary Files [file 41467_2019_8690_MOESM3_ESM.docx]

**Description of Supplementary Files**

**File Name:** Supplementary Video 1

**Description:** Fluorescence of mouse fundus 1.5-3 min after IV injection of NP-AMF.

**File Name:** Supplementary Video 2

**Description:** Fluorescence of mouse fundus 4-5 min after IV injection of NP-AMF.
